# *MTNR1B *rs10830963 is associated with fasting plasma glucose, HbA_1C _and impaired beta-cell function in Chinese Hans from Shanghai

**DOI:** 10.1186/1471-2350-11-59

**Published:** 2010-04-14

**Authors:** Chen Liu, Ying Wu, Huaixing Li, Qibin Qi, Claudia Langenberg, Ruth JF Loos, Xu Lin

**Affiliations:** 1 Key Laboratory of Nutrition and Metabolism, Institute for Nutritional Sciences, Shanghai Institutes for Biological Sciences, Chinese Academy of Sciences, Shanghai, China; 2 Graduate School of the Chinese Academy of Sciences, Shanghai, 200031, China; 3 MRC Epidemiology Unit, Institute of Metabolic Science, Addenbrooke's Hospital, Cambridge, UK

## Abstract

**Background:**

Genome-wide association studies (GWAS) in White Europeans have shown that genetic variation rs10830963 in melatonin receptor 1B gene (*MTNR1B*) is associated with fasting glucose and type 2 diabetes, which has also been replicated in several Asian populations. As a variant in the gene involved in the regulation of circadian rhythms, the effect of the variant on sleep status remains unknown. This study aimed to investigate the effects of *MTNR1B *rs10830963 on fasting glucose, type 2 diabetes and sleep status in Chinese Hans.

**Methods:**

*MTNR1B *rs10830963 was genotyped in a population-based cohort including 3,210 unrelated Chinese Hans from Beijing and Shanghai, and tested for associations with risk of type 2 diabetes, diabetes-related traits and sleep status.

**Results:**

We confirmed the associations of *MTNR1B *rs10830963 with fasting glucose (beta = 0.11 mmol/l, 95%CI [0.03, 0.18], *P *= 0.005), glycated hemoglobin (HbA_1c_) (beta = 0.07%, 95%CI [0.02,0.12], *P *= 0.004) and homeostasis model assessment of beta-cell function (HOMA-B) (beta = -5.01%, 95%CI [-8.24,-1.77], *P *= 0.003) in the Shanghai, but not in the Beijing subpopulation (*P *≥ 0.58). The effect size of *MTNR1B *rs10830963 on fasting glucose in Shanghai Chinese Hans was comparable to that reported from other Asian populations. We found no evidence of associations with type 2 diabetes (OR 1.05 [0.90-1.23], *P *= 0.54), homeostasis model assessment of insulin sensitivity (HOMA-S) (*P *= 0.86) or sleep status (*P *≥ 0.44).

**Conclusions:**

A common variant in *MTNR1B *was associated with fasting glucose, HbA_1C _and HOMA-B but not with sleep status in Chinese Hans from Shanghai, strengthening the role of *MTNR1B *rs10830963 in fasting glycemia and impaired beta-cell function.

## Background

There is growing evidence suggesting that circadian rhythms are closely linked to metabolic regulation, and dysregulation of circadian rhythms may increase diabetes risk [1]. Consistently, melatonin, a major regulator of circadian rhythms, has been shown to influence both insulin secretion and glucose homeostasis, and both melatonin secretion and circadian rhythm are impaired in type 2 diabetes patients [2]. It is therefore likely that melatonin may provide a link between circadian rhythms and glucose homeostasis.

The melatonin effects on sleep and circadian phase are mainly mediated by activation of its two receptors: melatonin receptor 1A (MT1) and melatonin receptor 1B (MT2) [3]. They are encoded by *MTNR1A *and *MTNR1B*, respectively, and both expressed in human pancreatic islets [4]. Receptor MT1 is mainly expressed in alpha cells while MT2 is predominantly expressed in beta cells and upregulated in pancreatic islets of type 2 diabetic patients [2,4,5], suggesting that MT2 receptor may play a role in insulin secretion and type 2 diabetes.

Recently, several large-scale genome-wide association analyses using data from more than ten genome-wide association scans identified common variants in or near the *MTNR1B *gene to be robustly associated with fasting glucose levels in populations of Europeans descent [6-8], with SNP rs10830963 showing the most significant association signal [7]. Several replication studies in European [5,9-11] and Asian populations [12,13] showed reproducible associations for *MTNR1B *rs10830963. A case-control study including 1165 case and 1105 control of Chinese Hans from Shanghai [13] confirmed the associations of *MTNR1B *rs10830963 with increased risk of type 2 diabetes and increasing fasting glucose, while another study in general Japanese and Sri Lankan populations [12] reported association between the variant and fasting glucose with effect sizes similar to those observed in Chinese Hans [13]. Further analyses showed that the *MTNR1B *variants were also significantly associated with increased risk of type 2 diabetes, and with increased fraction of glycated hemoglobin (HbA_1C_), reduced beta-cell function as estimated by homeostasis model assessment of beta-cell function (HOMA-B), but not with fasting insulin level or insulin sensitivity [6,7]. The risk G-allele of rs10830963 also predicted future type 2 diabetes in both the MPP (the Malmö Prevention Project) and Botnia prospective studies [5]. Furthermore, nondiabetic individuals carrying the risk G-allele showed increased expression of *MTNR1B *in pancreatic islets [5] and the *MTNR1B *risk G-allele has been suggested to increase risk of impaired fasting glycemia and type 2 diabetes through impaired insulin secretion [5,9,11,14]. These observations provide strong evidence for a role of *MTNR1B *in glucose homeostasis and type 2 diabetes.

The aim of this study was to examine whether the association previously reported for rs10830963 which located in the only intron of *MTNR1B *could be replicated in a population-based cohort including 3,210 unrelated Chinese Hans from Shanghai and Beijing. We also tested for a role of *MTNR1B *rs10830963 in sleep duration and quality in this population.

## Methods

### Study participants

The sample consists of 3,210 unrelated individuals (1,423 men and 1,787 women) from the Study on Nutrition and Health of Aging Population in China, a population-based study of non-institutionalized Chinese Hans aged 50 to 70 years from Beijing and Shanghai. The design of this study have been previously described in detail [15]. Type 2 diabetes was defined as fasting plasma glucose ≥7.0 mmol/L and/or previously diagnosed diabetic (424 type 2 diabetes: 37% screen-detected, 63% previously diagnosed). Normal fasting glucose (NFG) was defined as fasting glucose <5.6 mmol/L (100 mg/dL). Homeostasis model assessment of insulin sensitivity (HOMA-S) and beta-cell function (HOMA-B) was estimated by Levy's computer model, and BMI was calculated as weight (kg)/height^2 ^(m^2^). A questionnaire of six questions was used to evaluate sleep quantity and quality, including sleep duration and self-reported sleep disturbance during the past month, as well as siesta frequency. Informed written consent was obtained from the participants and the Institutional Review Board of the Institute for Nutritional Sciences approved the study protocol.

### Genotyping

The SNP rs10830963 was genotyped by the GenomeLab SNPstream Genotyping System (Beckman Coulter) with 98.1% genotyping success rates. The concordance rate was 99.2% based on 12% duplicate samples (n = 384). The genotypic distributions were similar between Beijing and Shanghai (*P *= 0.11) and were in Hardy-Weinberg equilibrium (*P *= 0.28).

### Statistical methods

Generalized linear regression was used for the associations with diabetes-related quantitative traits and gene-geographical regions (Beijing/Shanghai) or gene-sleep status interactions in which participants with known diabetes or receiving glucose-lowering treatment (n = 276) were excluded. Logistic regression was applied to test the associations of rs10830963 with type 2 diabetes and sleep disorder. All the analyses above assumed an additive effect of the risk allele adjusting for age, sex, BMI and sleep status (where appropriate). The associations with sleep duration, siesta frequency and self-reported sleep disorder were carried out using nonparametric test (Kruskal-Wallis), χ^2 ^test and logistic regression, respectively. Given the significant interactions between the genotypes and the geographical regions on fasting glucose and HOMA-B, association studies were performed in Beijing and Shanghai subpopulations separately. We conducted meta-analyses applying Cochran Q test in Stata to evaluate the combined effect sizes of subpopulations and the heterogeneity of effect size across different ethnic populations. The meta-analyses were carried out using inverse variance weighting and random model. HOMA-S was log-transformed before analyses. Power calculations were assessed using Quanto software http://hydra.usc.edu/gxe. Statistical analyses were conducted using R (version 2.6.1) and Stata 9.2 (StataCorp, college station, Texas). Two-side *P *values < 0.05 were considered to be statistically significant.

## Results

The risk G allele frequency of *MTNR1B *rs10830963 in this Chinese population (42.3%, Beijing/Shanghai: 41.2%/43.5%) was comparable to that reported by the International HapMap CHB sample (47.8%), and higher than that for the CEU sample (30%). The characteristics of the study participants were summarized in Table 1.

**Table 1:** Characteristics of the study population

	Beijing	Shanghai	Total population	*P*
N (%male)	1574 (45.2)	1636 (43.5)	3210 (44.3)	
Age (years)	58.3 ± 5.9	58.9 ± 6.0	58.6 ± 6.0	0.0095
BMI (kg/m^2^)	25.1 (22.8-27.4)	23.5 (21.3-25.9)	24.2 (22.0-26.6)	< 0.0001
Current smoker (%)	499 (31.7)	403 (24.7)	902 (28.1)	< 0.0001
Current drinker (%)	584 (37.1)	327 (20.0)	911 (28.4)	< 0.0001
Physical activity, (MET-h/wk)	49.1 (23.1-76.4)	39.6 (23.1-65.5)	46.2 (23.1-69.3)	< 0.0001
Sleep duration, hour	8.0 (7.0-8.5)	7.5 (6.5-8.0)	8.0 (7.0-8.0)	< 0.0001
Self-reported sleep disorder (%)	362 (22.97)	251 (15.36)	613 (19.10)	< 0.0001
Fasting glucose (mmol/l)	6.16 ± 1.96	5.53 ± 1.42	5.84 ± 1.74	< 0.0001
HbA_1C _(%)	6.08 ± 1.22	5.90 ± 0.96	5.99 ± 1.10	< 0.0001
HOMA-B (%)	100.1 ± 44.9	120.0 ± 46.9	110.3 ± 47.0	< 0.0001
HOMA-S (%)	64.0 (47.3-89.5)	63.5 (46.9-85.1)	63.7 (47.1-86.9)	0.05
Diabetes (%)	272 (17.3)	152 (9.3)	424 (13.2)	< 0.0001
Obesity (%)	320 (20.3)	154 (9.4)	474 (14.8)	< 0.0001
Hypertension (%)	978 (62.1)	803 (49.1)	1781 (55.5)	< 0.0001

Data are mean ± SD, median (inter-quartile range) or n (%). *P *represents significance of the differences between Beijing and Shanghai subpopulations.

**Figure 1:**
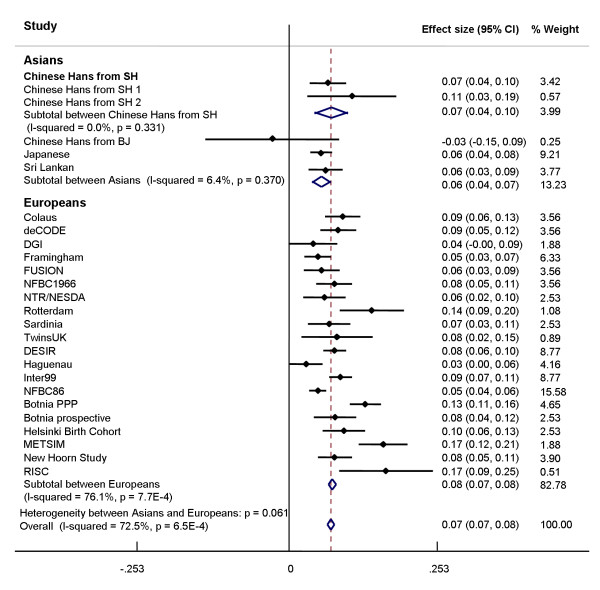
**Association of rs10830963 with fasting glucose under an additive model using meta-analyses**. The data in Asian populations are from the studies in Chinese (Chinese Hans from SH 1 [13], Chinese Hans from SH2 and BJ in this population), Japanese [12] and Sri Lankan [12], and data in Europeans are from studies of CoLaus [7], deCODE [7], DGI [7], Framingham [7], FUSION [7], NFBC1966 [7], NTR/NESDA [7], Rotterdam [7], Sardinia [7], TwinUK [7], DESIR [11], Haguenau [11], Inter99 [11], NFBC86 [11], Botnia PPP [5], Botnia prospective [5], Helsinki Birth Cohort [5], METSIM [5], New Hoorn Study [10] and RISC [9]. The black circles and horizontal lines represent the point estimated beta and 95% CI for of each study, respectively. The overall 95% CI for the meta-analyses is represented by diamond. SH, Shanghai; BJ, Beijing.

Consistent with the previous findings in White Europeans [5,7,11,14] and Asian populations [12,13], the *MTNR1B *rs10830963 G allele showed significant associations with increased levels of fasting glucose (beta = 0.11 mmol/l, 95%CI [0.03, 0.18], *P *= 0.005) and HbA_1C _(beta = 0.07%, 95%CI [0.02,0.12], *P *= 0.004) and with decreased HOMA-B values (beta = -5.01%, 95%CI [-8.24,-1.77], *P *= 0.003) in the Shanghai subpopulation, but not in the Beijing subpopulation (All traits *P *≥ 0.58) (*P *for interaction = 0.05 for fasting glucose, 0.09 for HbA_1C_, 0.01 for HOMA-B) (Table 2). The observed effect size on fasting glucose in Shanghai participants of this study was similar to that reported for Shanghai Chinese Hans by Ronn *et al. *[13] (*P *for heterogeneity = 0.33). Meta-analyses of our data in Chinese and the published data, including all studies in White Europeans [5,7,9-11] and Asians [12,13], showed significant heterogeneity in effect size on fasting glucose levels among white Europeans (*P *for heterogeneity = 7.7 × 10^-4^) but not among Asian populations (*P *for heterogeneity = 0.37). The overall effect size in Asians (0.06 mmol/l/per allele) tended (*P *for heterogeneity = 0.06) to be smaller than that in White Europeans (0.08 mmol/l/per allele) (Figure 1)."

**Table 2:** Associations of rs10830963 with fasting glucose, HbA_1C_, HOMA-B and HOMA-S.

*MTNR1B *rs10830963	Beijing (1389)		Shanghai (1497)		Total population (2886)		*P *for Interaction		
	
	beta (SE)	*P*_add_	beta (SE)	*P*_add_	beta (SE)	*P*_add_	SNP × Region	SNP × Sleep duration	SNP × Sleep disorder
Glucose(mmol/l)									
Model 1	-0.03 (0.06)	0.59	0.11 (0.04)	0.005	0.04 (0.07)	0.51	0.05	0.35	0.48
Model 2	-0.03 (0.05)	0.58	0.11 (0.04)	0.005	0.04 (0.07)	0.52			
Model 3	-0.03 (0.06)	0.59	0.11 (0.04)	0.005	0.04 (0.07)	0.51			
HbA_1C _(%)									
Model 1	-0.003 (0.04)	0.93	0.07 (0.02)	0.004	0.04 (0.04)	0.30	0.09	0.52	0.22
Model 2	-0.004 (0.04)	0.92	0.07 (0.03)	0.004	0.04 (0.04)	0.31			
Model 3	-0.004 (0.04)	0.93	0.07 (0.03)	0.004	0.04 (0.04)	0.30			
HOMA-B (%)									
Model 1	0.88 (1.58)	0.58	-5.01 (1.65)	0.003	-2.04 (2.94)	0.49	0.01	0.75	0.34
Model 2	0.91 (1.57)	0.56	-5.01 (1.65)	0.003	-2.02 (2.96)	0.49			
Model 3	0.88 (1.58)	0.58	-5.01 (1.65)	0.003	-2.04 (2.94)	0.49			
Log-transformed	HOMA-S (%)								
Model 1	-0.004 (0.02)	0.82	0.008 (0.02)	0.66	0.002 (0.01)	0.86	0.65	0.31	0.73
Model 2	-0.005 (0.02)	0.82	0.008 (0.02)	0.66	0.002 (0.01)	0.86			
Model 3	-0.004 (0.02)	0.82	0.008 (0.02)	0.66	0.002 (0.01)	0.86			

Participants previously diagnosed with type 2 diabetes or receiving glucose-lowering treatment (n = 264) were excluded from the analyses. HOMA-S values were log-transformed before analyses. *P *values and beta (SE) for Beijing and Shanghai subpopulations were calculated using linear regression model assuming an additive model. The combined *P *values and beta (SE) were calculated in meta-analyses using random model.

Model 1: Adjusted for age, sex and BMI.

Model 2: Adjusted variables in model 1 plus sleep duration.

Model 3: Adjusted variables in model 1 plus self-reported sleep disorder.

*P *values for interaction were tested in linear regression model using terms of SNP × Region (Beijing/Shanghai), SNP × Sleep duration or SNP × Sleep disorder.

We observed no significant associations with log-transformed HOMA-S (*P *≥ 0.66) and type 2 diabetes (ORs: 0.99-1.14, *P *≥ 0.31) either in Shanghai and Beijing subpopulations or in the whole population. No significant associations of *MTNR1B *rs10830963 with sleep duration, siesta frequency or sleep disorder were found in either Beijing or Shanghai subpopulations (*P *≥ 0.44) (Table 3). Moreover, further adjustment for sleep duration (Table 2, model 2) or self-reported sleep disorder (Table 2, model 3) did not materially change the associations for diabetes-related quantitative traits in either Shanghai or Beijing subpopulation, and no significant gene-sleep status interaction (*P *for interaction ≥ 0.22) was observed (Table 2).

**Table 3:** Association of rs10830963 with sleep status

*MTNR1B *rs10830963	Beijing				Shanghai			
	
	CC	CG	GG	*P*	CC	CG	GG	*P*
N (%male)^a^	539 (46.9)	747 (45.0)	265 (42.3)	0.45	500 (44.6)	807 (42.4)	292 (45.2)	0.61
Age (years)^b^	58.5 (6.0)	58.2 (5.8)	58.5 (6.1)	0.55	58.8 (6.2)	58.8 (5.9)	59.3 (6.1)	0.49
BMI (kg/m^2^)^b^	25.4 (3.7)	25.2 (3.6)	25.0 (3.7)	0.36	23.7 (3.3)	23.5 (3.3)	23.8 (3.3)	0.51
Sleep duration (hour)^c^	8.0 (7.0-8.3)	8.0 (7.0-8.5)	8.0 (7.0-8.0)	0.95	7.5 (6.5-8.0)	7.5 (6.0-8.0)	7.8 (6.5-8.0)	0.44
Siesta frequency (%)^a^								
Never	157 (29.1)	192 (25.7)	73 (27.6)	0.92	262 (52.4)	402 (49.8)	155 (53.1)	0.81
Occasional	87 (16.1)	122 (16.3)	43 (16.2)		92 (18.4)	171 (21.2)	54 (18.5)	
Regularly during summer	63 (11.7)	96 (12.9)	32 (12.1)		62 (12.4)	102 (12.6)	40 (13.7)	
Regularly during the year	232 (43.1)	337 (45.1)	117 (44.1)		84 (16.8)	132 (16.4)	43 (14.7)	
Self-reported sleep disorder (%)^a^	127 (23.6)	164 (22.0)	67 (25.3)	0.50	77 (15.4)	126 (15.6)	42 (17.1)	0.88
Per-allele effect on self-reported sleep disorder (OR [95% CI])^d^	1.00 (0.85-1.19)			0.96	0.97 (0.79-1.18)			0.75

Data are mean ± SD, median (inter-quartile range), n (%), or odd ratio (95%CI).

*P *values were calculated using *χ*^2 ^test ^a^, linear regression model ^b^, Kruskal-Wallis test ^c^, or logistic regression model under an additive model adjusting for age, sex and BMI.

## Discussion

In this population-based sample of Chinese Hans, we confirmed that *MTNR1B *rs10830963 G allele was significantly associated with increased fasting plasma glucose and HbA_1C _in Shanghai, but not in Beijing Hans. These findings in the Shanghai subpopulation were consistent with those of previous studies [5,7,11-14] and further highlight the importance of *MTNR1B *rs10830963 variant for insulin secretion. Significant heterogeneity in effect size on fasting glucose was observed only among White Europeans, but not among Asians in meta-analyses with data from our study and the previously reported. The overall effect size on fasting glucose among Asians tended to be smaller than that in white Europeans. However, this result should be interpreted with caution since the heterogeneity observed in White Europeans may introduce bias to the comparison among Chinese and European overall effects. More studies are required to draw a firm conclusion.

To explore the possible reason for the discrepancies between Beijing and Shanghai, we compared their difference in allele frequencies, prevalence of type 2 diabetes and environmental/lifestyle factors. Yet, the G-allele frequencies and genotype distributions of the *MTNR1B *rs10830963 were similar between Beijing and Shanghai subpopulations. Given that individuals from Beijing have a higher prevalence of type 2 diabetes than their Shanghai counterparts and also live a less healthy lifestyle (Table 1), the discrepancies observed between Beijing and Shanghai participants could possibly be attributed to unmeasured environmental/lifestyle factors that may have masked the effect of the *MTNR1B *variant.

Although we failed to replicate the association for type 2 diabetes risk in this study, the observed effect size was within the 95% confidence interval of the effect size observed for White Europeans [6,7]. Assuming an additive model and a minor allele frequency of 42.3%, we had less than 35% power to detect the previously reported OR of 1.09 [7] for type 2 diabetes and beta value of 0.072 mmol/l [7] for fasting glucose at *P *< 0.05. Thus, absence of association with type 2 diabetes and related quantitative traits in our study could be due to insufficient power.

Considering the important role of melatonin in modulating circadian rhythms and sleep duration, the association of *MTNR1B *rs10830963 with fasting plasma glucose and insulin secretion might be partially explained by the disrupted sleep and circadian rhythms in the carriers of risk G allele. However, our study provided no evidence for the association with sleep duration, siesta frequencies or self-reported sleep disorder. Also, further adjustment for these sleep variables did not materially change the associations with HOMA-B, fasting plasma glucose and HbA_1C_. Our study had 80% power to detect odd ratio (OR) ≥ 1.27, 1.32 and 1.20 for self-reported sleep disorder at a significance of 5% in Beijing, Shanghai subpopulation and in whole population, respectively. Since the effect size of this variant on sleep status is largely unknown, more studies with larger sample size may be required before the conclusion that sleep duration or quality has no impact on the association between *MTNR1B *rs10830963 and diabetes-related traits can be drawn.

## Conclusions

We confirmed the associations of *MTNR1B *rs10830963 with fasting glucose, HbA_1C _and HOMA-B in Chinese Hans from Shanghai. These associations were not affected by sleep duration or quality, but more studies with larger sample size are needed to verify this.

## Competing interests

The authors declare that they have no competing interests.

## Authors' contributions

CL drafted the manuscript. YW carried out SNP genotyping, data collection and results interpretation. HL supervised and co-drafted the manuscript. QQ has been involved in data collection and results interpretation. CL and RJFL have contributed to the scientific ideas, as well as supervised and revised the manuscript critically. XL, as the principal investigator for the project of the Nutrition and Health of Aging Population in China, contributed to the scientific ideas, grant application, experiment design, result interpretation and revised the manuscript critically. All authors have read and approved the manuscript.

## Pre-publication history

The pre-publication history for this paper can be accessed here:

http://www.biomedcentral.com/1471-2350/11/59/prepub
